# Connexin and pannexin (hemi)channels in the liver

**DOI:** 10.3389/fphys.2013.00405

**Published:** 2014-01-10

**Authors:** Michaël Maes, Elke Decrock, Bruno Cogliati, André G. Oliveira, Pedro E. Marques, Maria L. Z. Dagli, Gustavo B. Menezes, Gregory Mennecier, Luc Leybaert, Tamara Vanhaecke, Vera Rogiers, Mathieu Vinken

**Affiliations:** ^1^Department of Toxicology, Center for Pharmaceutical Research, Vrije Universiteit BrusselBrussels, Belgium; ^2^Physiology Group, Department of Basic Medical Sciences, Ghent UniversityGhent, Belgium; ^3^Department of Pathology, School of Veterinary Medicine and Animal Science, University of Sao PauloSao Paulo, Brazil; ^4^Department of Morphology, Institute of Biological Sciences, Universidade Federal de Minas GeraisBelo Horizonte, Brazil

**Keywords:** connexin, pannexin, hemichannel, gap junction, hepatocyte, cell death, inflammation

## Abstract

The liver was among the first organs in which connexin proteins have been identified. Hepatocytes harbor connexin32 and connexin26, while non-parenchymal liver cells typically express connexin43. Connexins give rise to hemichannels, which dock with counterparts on adjacent cells to form gap junctions. Both hemichannels and gap junctions provide pathways for communication, via paracrine signaling or direct intercellular coupling, respectively. Over the years, hepatocellular gap junctions have been shown to regulate a number of liver-specific functions and to drive liver cell growth. In the last few years, it has become clear that connexin hemichannels are involved in liver cell death, particularly in hepatocyte apoptosis. This also holds true for hemichannels composed of pannexin1, a connexin-like protein recently identified in the liver. Moreover, pannexin1 hemichannels are key players in the regulation of hepatic inflammatory processes. The current paper provides a concise overview of the features of connexins, pannexins and their channels in the liver.

## Introduction

**Figure 1:**
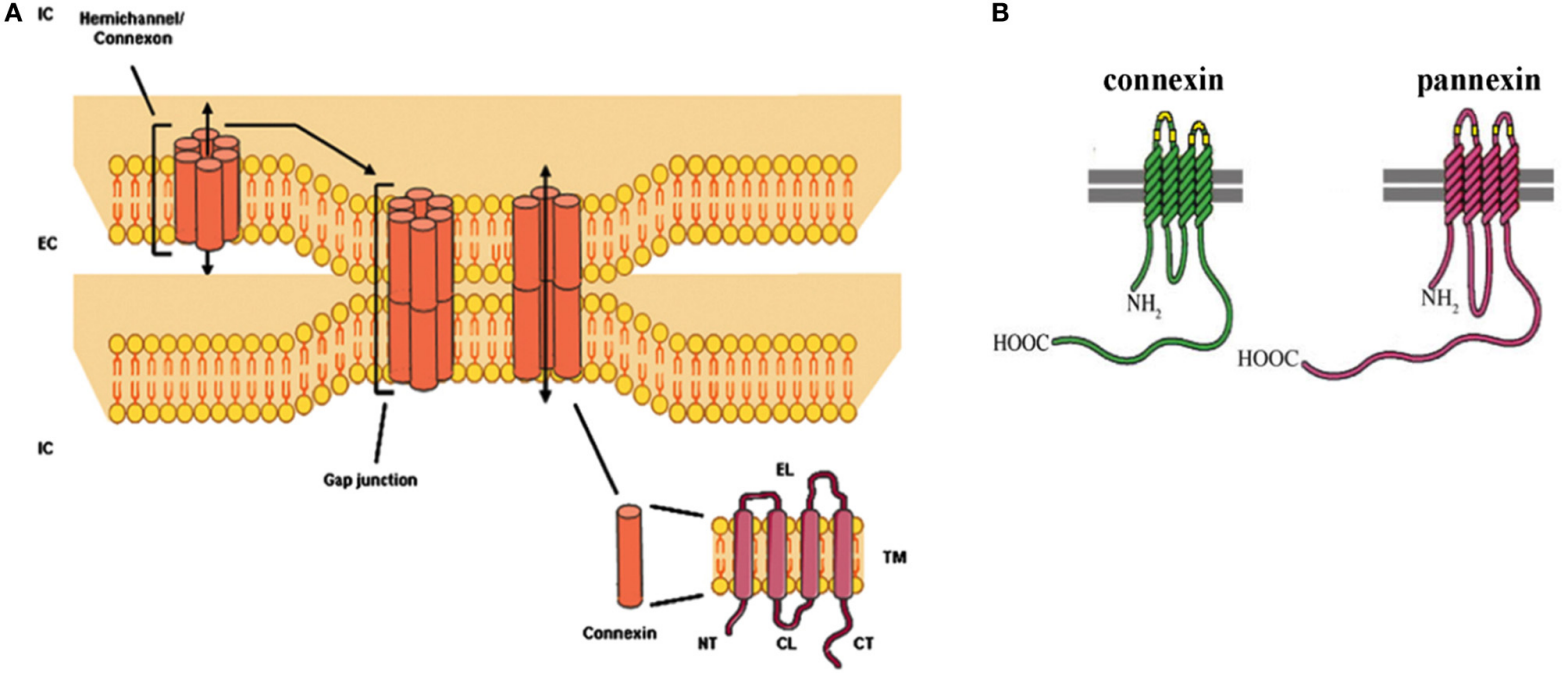
**(A)** *Molecular architecture of gap junctions*. Gap junctions are grouped in plaques at the cell plasma membrane surface of 2 adjacent cells and are composed of 12 connexin proteins organized as 2 hexameric hemichannels. The connexin protein consists of 4 membrane-spanning domains (TM), 2 extracellular loops (EL), 1 cytoplasmic loop (CL), 1 cytoplasmic aminotail (NT) and 1 cytoplasmic carboxytail (CT) (EC, extracellular; IC, intracellular) (Vinken et al., 2008; Decrock et al., 2009). **(B)** *Structural comparison of connexins and pannexins* (Chekeni et al., 2010).

The liver is a unique organ endowed with a plethora of specialized functions and a strong regenerative capacity. The establishment of communicative networks between the different liver cell types is therefore indispensable. Non-parenchymal liver cells preferentially have paracrine or juxtacrine contacts amongst themselves and with other hepatic cell types (Kmieć, 2001). Hepatocytes, the most prominent liver cell population, directly communicate with each other through gap junctions. The latter are formed by head-to-head docking of 2 hemichannels of neighboring cells, which in turn are composed of 6 connexin (Cx) proteins (Figure 1) (Vinken et al., 2006, 2008, 2009, 2010a,b, 2011; Decrock et al., 2009, 2011). Historically, these hemichannels have been considered as merely structural precursors of gap junctions. In the last decade, an accumulating body of evidence points to independent roles for hemichannels in cellular signaling by connecting the intracellular compartment with the extracellular environment (Vinken et al., 2006, 2010b; Decrock et al., 2009, 2011). More recently, a novel class of connexin-like proteins, the pannexin (Panx) proteins, has been identified. They assemble in a hemichannel configuration and are also named “single membrane channels” instead of hemichannels, as they do not appear to form gap junctions (D'Hondt et al., 2011; Bennett et al., 2012; Dahl and Keane, 2012; Wang et al., 2013a). In contrast to gap junctions, connexin and pannexin hemichannels seem to be mainly involved in pathological processes, including cell death and inflammation (Decrock et al., 2009; D'Hondt et al., 2009; Chekeni et al., 2010; Ganz et al., 2011; Bennett et al., 2012). These emerging roles for connexin and pannexin hemichannels are discussed in the present paper with focus on their relevance to liver (dys)functionality. Furthermore, an updated overview of the currently available knowledge regarding hepatic connexin and pannexin expression as well as liver gap junctions is provided.

## Connexins and pannexins in the liver

**Figure 2:**
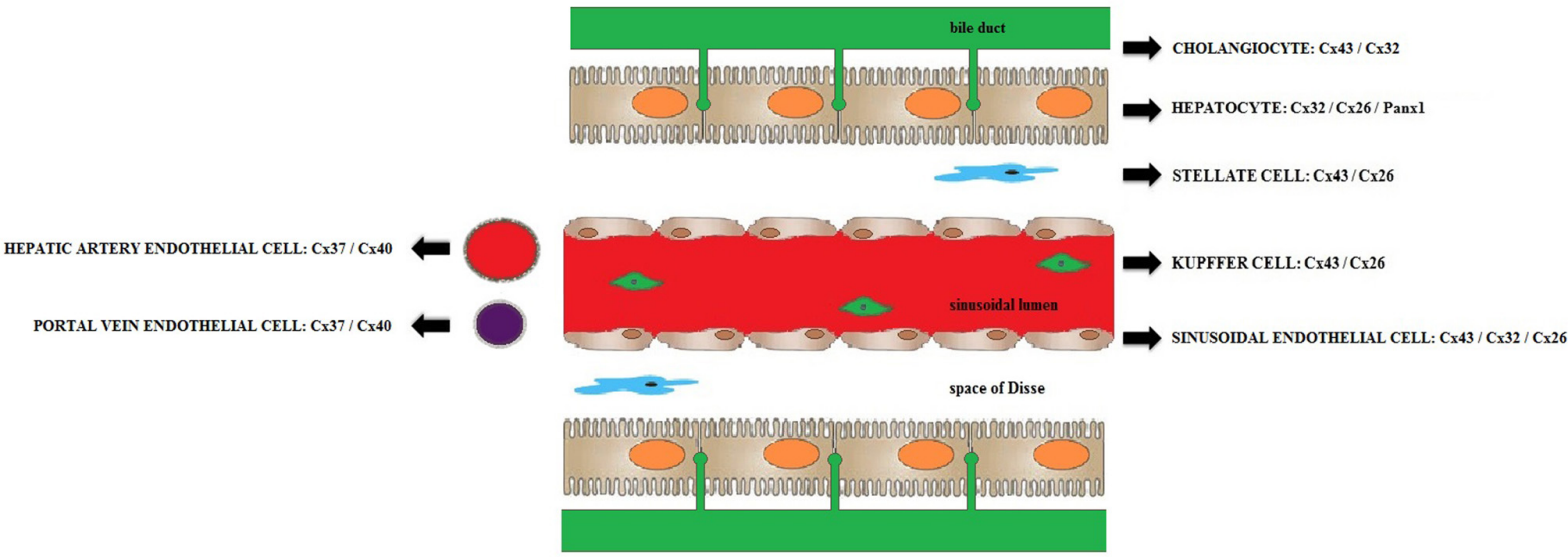
**Connexins and pannexins expressed in the liver**. Schematic overview of the most relevant connexin and pannexin species expressed in rodent and human livers (Kumar and Gilula, 1986; Paul, 1986; Nicholson et al., 1987; Zhang and Nicholson, 1989; Chaytor et al., 2001; Bode et al., 2002; Fischer et al., 2005; Shiojiri et al., 2006; Xiao et al., 2012).

At present, more than 20 connexin proteins have been identified in human beings and rodents. They all share a similar structure consisting of 4 membrane-spanning domains, 2 extracellular loops, a cytoplasmic loop, and cytosolic N-terminal and C-terminal regions (Figure 1). The different connexin family members are typically named after their molecular weight expressed in kilodaltons (Decrock et al., 2009; Vinken et al., 2009). Hepatocytes express Cx32 and to a lesser extent Cx26, which represents about 90 and 5%, respectively, of the total connexin amount in rat and human livers. In contrast, most non-parenchymal liver cells, including stellate cells and Kupffer cells, mainly harbor Cx43, while liver vascular cells predominantly express Cx37 and Cx40 (Figure 2) (Kumar and Gilula, 1986; Paul, 1986; Nicholson et al., 1987; Zhang and Nicholson, 1989; Chaytor et al., 2001; Bode et al., 2002; Fischer et al., 2005; Shiojiri et al., 2006). A typical hallmark of the liver includes zonation, which is also manifested at the connexin level. Indeed, Cx32 is uniformly distributed throughout the liver, whereas Cx26 is preferentially expressed in the periportal acinar area (Berthoud et al., 1992; Kojima et al., 1995; Iwai et al., 2000). Liver connexin expression patterns undergo drastic changes upon both differentiation (i.e., liver organogenesis) and dedifferentiation (i.e., liver disease) (Vinken, 2012; Vinken et al., 2008, 2012a). Specifically, early hepatic progenitor cells switch from Cx43 to Cx26, but especially to Cx32 during differentiation into hepatocytes (Zhang and Thorgeirsson, 1994; Neveu et al., 1995; Naves et al., 2001; Paku et al., 2004). An inverse process is observed when chronic liver disease, such as liver fibrosis and cirrhosis, progressively turns into liver cancer (Vinken, 2012). Moreover, connexin expression and subcellular localization are significantly altered during liver cell growth (Traub et al., 1983; Kren et al., 1993; Temme et al., 2000). Proliferation is a rare event in the adult liver, but upon partial hepatectomy, the remaining liver lobes start to grow and the original size becomes restored within a week (Taub, 2004). Cx32 expression was found to increase during early hepatocyte cell cycling, followed by a sharp decline (Traub et al., 1983; Dermietzel et al., 1987; Sugiyama and Ohta, 1990; Miyashita et al., 1991; Kren et al., 1993; Temme et al., 2000; Kojima et al., 2003). Less consistent changes have been reported for Cx26 (Kren et al., 1993; Temme et al., 2000). Furthermore, Cx37 and Cx40 are clearly upregulated, while Cx43 tends to stay unaffected during liver regeneration (Kren et al., 1993).

Only 3 pannexin proteins have yet been characterized. They show structural similarity with connexin proteins, though pannexins typically have longer C-terminal regions and extracellular loops (D'Hondt et al., 2011; Wang et al., 2013a). Unlike connexins, very little is known about the occurrence of pannexins in the liver, although a number of studies have demonstrated the presence of Panx1 (Bruzzone et al., 2003; Csak et al., 2011; Ganz et al., 2011). In a more recent study, Panx1 mRNA was detected in cultured rat primary hepatocytes as well as in rat and human cancerous hepatocytes (Xiao et al., 2012). A single study also showed expression of Panx2 in the lateral plasma membrane fraction of primary rat hepatocytes (Li et al., 2008).

## Gap junctions in the liver

In the liver, the vast majority of gap junctions are formed between hepatocytes. They occupy as much as 3% of the hepatocellular membrane surface and form gap junctional plaques ranging from 0.2 to 1 μm in diameter and containing from 10 to more than 10,000 gap junction channels. Hepatocellular gap junctions in the pericentral acinar area mainly consist of homomeric hemichannels (i.e., containing Cx32), whereas their periportal counterparts can also be built up by heteromeric hemichannels (i.e., composed of both Cx26 and Cx32) (Vinken et al., 2008). Cx32 in the liver interacts with several other junctional proteins, such as occludin and claudin1 (Kojima et al., 2001), but also with mitochondrial proteins, including sideroflexin1 (Fowler et al., 2013). Gap junctions provide a generic pathway for communication between adjacent cells, called gap junctional intercellular communication (GJIC). GJIC includes the passive diffusion of small and hydrophilic molecules, such as cyclic adenosine monophosphate, adenosine 5' triphosphate (ATP), inositol triphosphate (IP3) and ions (Alexander and Goldberg, 2003; Dbouk et al., 2009; Decrock et al., 2009; Vinken et al., 2011). Homotypic Cx26 gap junctions favor cation transfer, whereas homotypic Cx32 gap junctions promote anion passage (Bukauskas et al., 1995). Likewise, ATP passes significantly better through gap junctions formed by Cx43 compared to Cx32-based channels (Goldberg et al., 2002).

Hepatocellular gap junctions are indispensable for maintaining the metabolic competence of the liver. In particular, Cx32-based GJIC underlies a number of liver-specific functions, including glycogenolysis (Stumpel et al., 1998), albumin secretion (Yang et al., 2003), bile secretion (Temme et al., 2001), ammonia detoxification (Yang et al., 2003) and xenobiotic biotransformation (Neveu et al., 1994; Shoda et al., 1999, 2000). Curiously, genetic ablation of Cx26 and Cx32 in mice does not drastically alter basal liver functionality (Ott et al., 2006). In line with this finding, Cx26 gene mutations in humans have been associated with skin diseases and deafness, but not with abnormalities in the liver (Lee and White, 2009), yet these patients display a gain of Cx26 hemichannel function (Mhaske et al., 2013). Similarly, human Cx32 gene mutations typically lead to neurological disorders, while leaving the liver largely unaffected (Abrams et al., 2000). However, Cx32-deficient mice, unlike their Cx26-lacking counterparts (Marx-Stoelting et al., 2008), have been found more susceptible to spontaneously occurring and chemically induced hepatocarcinogenesis (Temme et al., 1997).

The biochemical identity of the messengers that travel through hepatocyte gap junctions and that affect liver functionality remains largely elusive, though an exception exists for glycogenolysis. Breakdown of glycogen to glucose is triggered by hormonal and neuronal stimuli and predominantly occurs at the periportal acinar pole. Pericentral hepatocytes also show glycogenolytic activity, albeit to a lesser extent (Stumpel et al., 1998; Saez et al., 2003). Cx32-based gap junctions therefore drive the propagation of the glycogenolytic response from the periportal to the pericentral area. In fact, they facilitate the intercellular exchange of IP3, which activates calcium release from endoplasmic reticulum stores, in turn evoking calcium waves throughout the acinar tract (Clair et al., 2001; Saez et al., 2003; Gaspers and Thomas, 2005). Indeed, Cx32 knock-out mice display lowered blood glucose levels upon glycogenolytic stimulation (Nelles et al., 1996; Stumpel et al., 1998). Similarly, the spread of calcium waves through Cx43-based gap junctions controls ductular secretion from cholangiocytes and thus bile formation (Nathanson et al., 1999; Bode et al., 2002).

GJIC is clearly involved in liver cell growth. However, the exact role and overall relevance of gap junctions in hepatocyte cell cycling is matter of debate. In the regenerating liver of rats treated with an inhibitor of the mitogen-activated protein kinase pathway, the reduction of Cx32 production is counteracted, with no effects on hepatocyte proliferative activity (Kojima et al., 2003). This indicates that downregulation of GJIC may occur independently of hepatocyte proliferation. In the regenerating liver of Cx32 knock-out mice, hepatocellular proliferative activity is not affected, but the extent of synchronous initiation and termination of DNA synthesis is decreased (Kojima et al., 1997; Temme et al., 2000). Based on this observation, reduction of GJIC does not provide a direct signal for hepatocytes to divide, but rather permits cell cycle progression upon mitogenic stimulation. On the other hand, a plethora of studies have shown determinate functions for gap junctions in liver cell proliferation control, rather than merely an assisting role in growth progression (Koffler et al., 2000; Ruch, 2000; Yano et al., 2001). It should be stressed, however, that such studies typically rely on genetic ablation of connexin expression, thus impeding discrimination between GJIC and connexin hemichannel communication.

## Connexin and pannexin hemichannels in the liver

Although still surrounded by a lot of controversy, it is now accepted that connexin hemichannels autonomously establish a pathway for cellular signaling between the cytosol of individual cells and their extracellular environment (Decrock et al., 2009; Chandrasekhar and Bera, 2012; Kar et al., 2012). The messengers that diffuse through connexin hemichannels are very similar to those that can permeate gap junctions and typically include ATP, glutamate and glutathione. However, in contrast to gap junctions, connexin hemichannels have a low probability to be open. They can be opened by a number of stimuli that are of pathological origin, such as changes in extracellular or intracellular calcium concentration, oxidative stress, induced metabolic inhibition, ischemia/reperfusion insults and inflammatory conditions. Hence, connexin hemichannels are frequently referred to as pathological pores (Decrock et al., 2009; Chandrasekhar and Bera, 2012; Kar et al., 2012). This has been well exemplified in the context of cell death. Although a limited set of reports described cytoprotective functions for connexin hemichannels (Plotkin et al., 2002; Okuda et al., 2013), most scientific evidence available points to pro-active roles for connexin hemichannels in the cell death process, involving the formation of a toxic pore or contributing to a paracrine cell death pathway (Kalvelyte et al., 2003; Takeuchi et al., 2006; Ramachandran et al., 2007; Decrock et al., 2009). Connexin hemichannels not only occur at the plasma membrane surface, but also reside at other subcellular locations, such as the mitochondria, where they have been linked to cell survival (Goubaeva et al., 2007; Lu et al., 2010; Azarashvili et al., 2011; Trudeau et al., 2012; Fowler et al., 2013). In this context, Cx43 translocates to the mitochondria where it interacts with Bax to initiate the mitochondrial apoptotic pathway in pancreatic cancer cells (Sun et al., 2012). Furthermore, mitochondrial Cx43 plays a role in myocardial ischemia-reperfusion injury by interfering with reactive oxygen species signaling (Ruiz-Meana et al., 2008) and facilitates ATP production (Boengler et al., 2012). More recently, mitochondrial Cx43-based hemichannels were found to assist in mitochondrial potassium uptake (Boengler et al., 2013).

Our group was the first to show the functional presence of connexin hemichannels in hepatocytes. Upon induction of Fas-mediated apoptosis in cultures of primary hepatocytes, GJIC rapidly declines, which is associated with a decay of the gap junctional Cx32 protein pool. At the same time, levels of newly synthesized Cx32 protein increase and gather in a hemichannel configuration. This becomes particularly evident toward the end stages of the cell death process (Vinken et al., 2010c). Subsequent experiments showed that Cx32-based hemichannels facilitate the apoptotic-to-necrotic transition in hepatocytes. Primary hepatocytes in culture are known to progressively lose their differentiated status, whereby Cx43 becomes *de novo* produced. Work with specific channel inhibitors demonstrated that Cx43 signaling, also involving hemichannels, underlies the onset of spontaneous apoptosis, which accompanies the dedifferentiation process in cultures of primary hepatocytes (Vinken et al., 2012b). In a more recent study, Cx43 production in cultured hepatocytes was epigenetically silenced followed by global protein and metabolite profiling. Among the proteins altered were several mitochondrial proteins. These data thus could further substantiate the existence of a mitochondrial connexin pool, and can be reconciled with a role for Cx43 signaling in spontaneously occurring apoptosis in primary hepatocyte cultures (Vinken et al., 2013).

Pannexin hemichannels have also been identified as mediators of apoptotic processes (Chekeni et al., 2010; Qu et al., 2011; Sandilos et al., 2012). Pannexin hemichannels can be opened by various pathological stimuli such as oxygen glucose deprivation, metabolic inhibition and S-nitrosylation (Thompson et al., 2006; Zhang et al., 2008). Similar to connexins (Yin et al., 2001; Theiss et al., 2007), Panx1 is an established target for caspases, which results in the formation of a constitutively open channel and the release of the so-called “find-me” signals, such as ATP and uridine 5′ triphosphate (UTP), at the earliest stages of cell death in order to recruit phagocytes (Chekeni et al., 2010; Sandilos et al., 2012). Both nucleotides act as “damage-associated molecular patterns,” which are released during immunogenic cell death and serve as a signal to initiate and amplify cell death as well as to induce inflammation (Elliott et al., 2009; Chekeni et al., 2010). In fact, Panx1 plays a major role in the regulation of inflammatory processes and thus in innate immunity. Panx1 is instrumental for activating the inflammasome, a multiprotein complex involved in innate immunity and caspase 1 activation, and subsequent processing and release of the pro-inflammatory cytokines interleukin 1 beta and interleukin 18. Activation of the inflammasome has been seen in lipopolysaccharide-stimulated macrophages (Pelegrin and Surprenant, 2006, 2007) as well as in astrocytes and neurons (Silverman et al., 2009). Further investigation demonstrated that Panx1 hemichannel opening, induced by ATP stimulation of P_2_X_7_ receptors (P_2_X_7_R), facilitates the entry of bacterial inflammatory signals into the cytosol (Kanneganti et al., 2007). Panx1 is known to co-localize with the P_2_X_7_R and to form a “death receptor” complex (Locovei et al., 2007). Here, prolonged stimulation of the P_2_X_7_R results in the opening of a non-selective pore that may correspond to the Panx1 hemichannel. Extracellular ATP acts on the P_2_X_7_R, leading to Panx1 hemichannel opening (Pelegrin and Surprenant, 2006, 2007; Locovei et al., 2007; Iglesias et al., 2008). This mechanism may also apply for connexins, since Cx43 and P_2_X_7_R were seen to co-localize and co-immunoprecipitate in mouse macrophages and J774 cells (Fortes et al., 2004). However, a more recent study based on the use of Panx1^−/−^ mice and P_2_X_7_R^−/−^ mice demonstrated that Panx1 and the P_2_X_7_R function in distinct signaling pathways. Whereas only the P_2_X_7_R was necessary for inflammatory responses in lipopolysaccharide-primed macrophages, Panx1 was dispensable in the migrating phagocyte but was essential for the release of the “find me” signals from apoptotic cells to recruit macrophages (Qu et al., 2011).

Treatment of mice with lipopolysaccharide, results in elevated levels of Panx1 in the liver. Also, an increased production of active interleukin 1 beta and interleukin 18 is observed under these circumstances (Ganz et al., 2011). In addition to driving the inflammasome, Panx1 contributes to pathophysiological ATP release in lipoapoptosis induced by saturated free fatty acids, a key morphologic and pathological feature of human non-alcoholic steatohepatitis. Using a rat liver cell line, it has been shown that saturated free fatty acids increase extracellular ATP concentrations. Extracellular ATP release as well as cytosolic uptake of an indicator dye were partly inhibited by suppressing Panx1 expression (Xiao et al., 2012). Thus, Panx1-based hemichannels may play an important role in hepatic inflammation by mediating an increase in extracellular ATP levels in lipotoxic liver injury.

## Conclusions and perspectives

Although still being in its infancy, it has become clear in the last few years that connexin and pannexin hemichannels fulfill critical functions in the regulation of cell death and inflammation. Only a handful of published reports have addressed these features in the liver and all of those rely on the use of *in vitro* settings (Decrock et al., 2009; D'Hondt et al., 2009; Chekeni et al., 2010; Ganz et al., 2011). Although some physiological roles have been attributed to hemichannels (Penuela et al., 2013), this remains to be demonstrated in the liver. It will be challenging in the upcoming years to evaluate the *in vivo* relevance of hepatic connexin and pannexin hemichannels. Several liver diseases, such as hepatitis, fibrosis and cholestasis, are associated not only with the onset of cell death and inflammation, but also with modifications in connexin expression patterns and activity (Vinken, 2012). In fact, as discussed in the current paper, it is conceivable to assume that connexin, but especially, pannexin hemichannels drive the process of hepatocyte cell demise by releasing “find me” signals to initiate their clearance (Chekeni et al., 2010). Furthermore, recent data suggest the critical involvement of Panx1 hemichannels in liver inflammation (Ganz et al., 2011; Xiao et al., 2012). The knowledge that will be gained in this respect in the upcoming years could open new perspectives for clinical therapy. Thus far, 2 studies have pinpointed the potential of interfering with hepatic connexin signaling in the clinical management of acute (Patel et al., 2012) and acute-on-chronic liver failure (Balasubramaniyan et al., 2013). However, these studies did not distinguish between GJIC and connexin hemichannel communication. Given the opposite roles of gap junctions and connexin hemichannels in the (dys)regulation of the homeostatic balance in the liver, such discrimination is key for a targeted and efficient clinical outcome. Specific and *in vivo*-applicable connexin hemichannel inhibitors have become available only very recently (Iyyathurai et al., 2013; Wang et al., 2013b). In this respect, a peptide called Gap19 was found to specifically block Cx43-based hemichannel signaling and to reduce cell death in a mouse model of cardiac ischemia/reperfusion insult (Wang et al., 2013c). Future efforts should be focused on the further development of such tools as well as on the testing of their clinical applicability, stressing the importance of the field of hepatology.

### Conflict of interest statement

The authors declare that the research was conducted in the absence of any commercial or financial relationships that could be construed as a potential conflict of interest.

## Abbreviations

ATP: adenosine 5′ triphosphate
Cx: connexin
GJIC: gap junctional intercellular communication
IP3: inositol triphosphate
P_2_X_7_R: P_2_X_7_ receptors
Panx: pannexin
UTP: uridine 5′ triphosphate.
